# A quantitative high-throughput screen identifies compounds that lower expression of the SCA2-and ALS-associated gene *ATXN2*

**DOI:** 10.1016/j.jbc.2022.102228

**Published:** 2022-07-02

**Authors:** Daniel R. Scoles, Mandi Gandelman, Sharan Paul, Thomas Dexheimer, Warunee Dansithong, Karla P. Figueroa, Lance T. Pflieger, Scott Redlin, Stephen C. Kales, Hongmao Sun, David Maloney, Robert Damoiseaux, Mark J. Henderson, Anton Simeonov, Ajit Jadhav, Stefan M. Pulst

**Affiliations:** 1 Department of Neurology, University of Utah, Salt Lake City, Utah, USA; 2 Division of Preclinical Innovation, National Center for Advancing Translational Sciences (NCATS), Rockville, Maryland, USA; 3 Department of Biomedical Informatics, University of Utah, Salt Lake City, Utah, USA; 4 Department of Molecular and Medical Pharmacology, Jonsson Comprehensive Cancer Center, California NanoSystems Institute, and Department of Bioengineering in the Samueli School of Engineering, University of California Los Angeles, Los Angeles, California, USA

**Keywords:** spinocerebellar ataxia type 2, ataxin-2, quantitative high-throughput screening, HSP90, HSP990, 17-DMAG, NaK-ATPases, cardiac glycoside, proscillaridin A, ASO, antisense oligonucleotide, ATP1A1/2/3, ATPase Na^+^/K^+^ transporting subunit alpha 1/2/3, ATXN2, ataxin-2, BAC, bacterial artificial chromosome, BiP, binding immunoglobulin protein, CDK, cyclin-dependent kinase, CHOP, C/EBP homologous protein, CMV, cytomegalovirus, 17-DMAG, 17-dimethylaminoethylamino-17-demethoxygeldanamycin, DMEM, Dulbecco’s modified Eagle's medium, DMSO, dimethyl sulfoxide, eIF2α, eukaryotic initiation factor 2α, ER, endoplasmic reticulum, HEK-293, human embryonic kidney 293 cell line, HSP, heat shock protein, HTS, high-throughput screening, KI, knock-in, LC3b, microtubule-associated protein 1A/1B-light chain 3b, MSSR, Molecular Screening Shared Resource, mTOR, molecular target of rapamycin, MTT, 3-(4,5-dimethylthiazol-2-yl)-2,5-diphenyltetrazolium, NCATS, National Center for Advancing Translational Sciences, NIH, National Institutes of Health, polyQ, polyglutamine, qHTS, quantitative high-throughput screen, qPCR, quantitative real-time PCR, *RLuc*, Renella luciferase, SCA2, spinocerebellar ataxia type 2, SG, stress granule, STAU1, Staufen 1, TDP-43, transactive response DNA-binding protein 43 kDa, UCLA, University of California, Los Angeles, UPR, unfolded protein response

## Abstract

CAG repeat expansions in the *ATXN2* (ataxin-2) gene can cause the autosomal dominant disorder spinocerebellar ataxia type 2 (SCA2) as well as increase the risk of ALS. Abnormal molecular, motor, and neurophysiological phenotypes in SCA2 mouse models are normalized by lowering *ATXN2* transcription, and reduction of nonmutant *Atxn2* expression has been shown to increase the life span of mice overexpressing the TDP-43 (transactive response DNA-binding protein 43 kDa) ALS protein, demonstrating the potential benefits of targeting *ATXN2* transcription in humans. Here, we describe a quantitative high-throughput screen to identify compounds that lower *ATXN2* transcription. We screened 428,759 compounds in a multiplexed assay using an ATXN2-luciferase reporter in human embryonic kidney 293 (HEK-293) cells and identified a diverse set of compounds capable of lowering *ATXN2* transcription. We observed dose-dependent reductions of endogenous ATXN2 in HEK-293 cells treated with procillaridin A, 17-dimethylaminoethylamino-17-demethoxygeldanamycin (17-DMAG), and heat shock protein 990 (HSP990), known inhibitors of HSP90 and Na^+^/K^+^-ATPases. Furthermore, HEK-293 cells expressing polyglutamine-expanded ATXN2-Q58 treated with 17-DMAG had minimally detectable ATXN2, as well as normalized markers of autophagy and endoplasmic reticulum stress, including STAU1 (Staufen 1), molecular target of rapamycin, p62, LC3-II (microtubule-associated protein 1A/1B-light chain 3II), CHOP (C/EBP homologous protein), and phospho-eIF2α (eukaryotic initiation factor 2α). Finally, bacterial artificial chromosome ATXN2-Q22 mice treated with 17-DMAG or HSP990 exhibited highly reduced ATXN2 protein abundance in the cerebellum. Taken together, our study demonstrates inhibition of HSP90 or Na^+^/K^+^-ATPases as potentially effective therapeutic strategies for treating SCA2 and ALS.

Spinocerebellar ataxia type 2 (SCA2) is a debilitating neurodegenerative disorder characterized primarily by gait ataxia, for which there are no disease-modifying treatments (1). SCA2 is caused by a CAG repeat expansion mutation in the *ATXN2* (ataxin-2) gene in an encoded region that results in polyglutamine (polyQ) expansion of the ATXN2 protein. In normal individuals, *ATXN2* usually contains 22 CAGs interrupted by one or two CAA codons, whereas SCA2 occurs when *ATXN2* contains pure CAG repeats numbering 33 or more (2). SCA2 is also characterized by anticipation where in families, CAG repeat lengths generally increase with each generation, and longer repeat lengths are associated with earlier age of onset and greater disease severity (3). SCA2 is included among nine total polyQ diseases, including dentatorubral-pallidoluysian atrophy, spinal-bulbar muscular atrophy, Huntington’s disease, and SCAs 1 to 3, 6, 7, and 17, each of which are characterized phenotypically by progressive neurodegeneration. Ataxia in SCA2 results from progressive loss of cerebellar Purkinje cells, and pathological defects in the brain stem are also observed, mainly involving the pontine and olivary nuclei. Targeting ATXN2 may also treat ALS: intermediate CAG repeat expansions increase ALS risk, and reduction of *Atxn2* expression in transactive response DNA-binding protein 43 kDa (TDP-43) transgenic mice increased life span and normalized TDP-43 aggregation (4, 5).

Since the discovery of *ATXN2* as the SCA2 gene in 1996 (3, 6, 7), we and others have made efforts to characterize ATXN2 function with the intention to identify therapeutic targets for SCA2. SCA2 is characterized by toxic gain-of-function mutations in the *ATXN2* gene. A principal *ATXN2* function is regulation of RNA processing, as the majority of known ATXN2-interacting proteins are RNA-binding proteins, including A2BP1/RBFox1, DDX6 (DEAD-box helicase 6), PABP1 (polyadenylate-binding protein 1), TDP-43, FUS (fused in sarcoma), and STAU1 (Staufen 1) (4, 8, 9, 10, 11, 12). ATXN2 also localizes to p-bodies and stress granules (SGs) supporting a role as a regulator of RNA translation or stability (8). We found that STAU1 and molecular target of rapamycin (mTOR) are both overabundant in SCA2 and ALS patient fibroblasts and mouse models associated with abnormal autophagy, which can be rescued by RNAi targeting *STAU1*, *ATXN2*, or *MTOR* (13). Transcriptomic profiling of cerebellar tissues from bacterial artificial chromosome (BAC) ATXN2-Q72 mice also showed the presence of severely reduced *Rgs8* (regulator of G protein signaling 8) mRNA levels as well as impaired *Rgs8* translation related to an abnormal interaction between polyQ-expanded ATXN2 and the *Rgs8* mRNA (14). *ATXN2* mutation is also associated with abnormal calcium homeostasis, and SCA2 Purkinje cells are characterized by elevated cytoplasmic Ca^2+^. This is caused in part by abnormal interaction by polyQ-expanded ATXN2 with inositol trisphosphate receptor resulting in increased Ca^2+^ release from the endoplasmic reticulum (ER) (15). However, elevated cytoplasmic Ca^2+^ may also result from impaired *Rgs8* expression since RGS proteins may inhibit mGlur1 (metabotropic glutamate receptor 1) (16), which is a positive regulator of Ca^2+^ release from internal stores (17).

Lowering *ATXN2* expression with antisense oligonucleotide (ASO) therapy and genetic interaction proved to be efficient in preclinical models of ALS and SCA2 (12, 18), and phase 1 trials are currently underway in ALS patients for the *ATXN2* ASO therapeutic BIIB105. In the present study, we sought to identify small molecules that could lower overall *ATXN2* expression with a quantitative high-throughput screen (qHTS). Using a cell line assay that reports *ATXN2* transcriptional activity, we identified multiple classes of compounds that effectively lowered *ATXN2* transcription, including heat shock protein 90 (HSP90) inhibitors and cardiac glycosides, which we further tested in cell cultures and BAC ATXN2-Q22 mice. These lead compounds could be modified by medicinal chemistry toward production of SCA2 and ALS therapeutics.

## Results

### Primary assay cell lines

**Figure 1:**
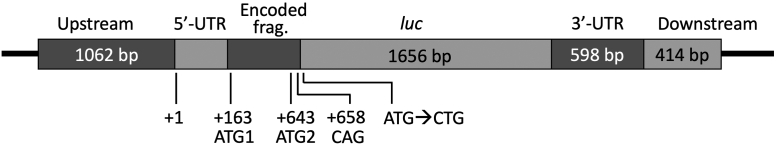
**Schematic representation of the ATXN2-luc expression cassette in plasmid pGL2h-5A3.***ATXN2* upstream sequence, the 5′-UTR, and a short encoded fragment are upstream of *luc*. Downstream of *luc* is the *ATXN2* 3′-UTR and additional downstream sequence. ATXN2, ataxin-2.

We produced six human embryonic kidney 293 (HEK-293) cell lines stably transfected to express ATXN2-luc (Fig. 1). Three were produced first including H1, H2, and H3, of which H2 expressed ATXN2-luc the greatest and was used in the University of California, Los Angeles (UCLA) Molecular Screening Shared Resource (MSSR) pilot screen. Subsequently, we produced an additional three lines designated S1, S2, and S3. Of all, we found that S2 expressed the greatest ATXN2-luc, and this line was used in the primary qHTS.

### MSSR pilot screen

We initially conducted a pilot screen of 65,728 compounds at the UCLA MSSR. H2 cells were treated with compounds for 24 h at a single 10 μM dose and then assayed for luciferase. Fig. S1 provides an example of the quality of the raw untransformed data in this pilot high-throughput screening (HTS). We observed 330 hits reducing *ATXN2*-luc expression by ≥2.7 SD, for an initial hit rate of 0.51%. We then rescreened 155 selected hit compounds at two compound doses (1 and 10 μM) using H2 cells and S1 cells and paired 3-(4,5-dimethylthiazol-2-yl)-2,5-diphenyltetrazolium (MTT) assays. Among these compounds, 43 were annotated, including 21 cardiac glycosides, six calcium channel blockers, three topoisomerase inhibitors, and one HSP90 inhibitor. Some of these annotated compounds also appeared as ATXN2-luc inhibitors in the primary qHTS (see later). Of the 155 compounds, we selected 12 that lowered ATXN2-luc by >2.7 SD but did not reduce cell viability (MTT assay) by >65% at either 1 μM or 10 μM, to further evaluate for modifying cytomegalovirus (CMV)-luc expression. Testing for CMV-luc modification determines performance of the assays for detecting compounds that function as either luciferase inhibitors or inhibitors of generalized transcription/translation. Among these 12 compounds, four compounds had activity specific against ATXN2-luc (ASN [Asinex] 5544420, ASN 5819045, ChemBridge 5228409, and ChemBridge 5718127). ChemBridge 5553825 was a potent CMV-luc inhibitor that was later used for further assessing the quality of the S2 primary screening cell line (see later). We performed multiconcentration testing of ChemBridge 5228409 and ChemBridge 5718127 using ATXN2-luc and CMV-luc, the primary and counterscreens, respectively, with matched MTT assays performed for viability assessment, demonstrating significant dose-dependent reduction of ATXN2-luc for ChemBridge 5718127. We did not further evaluate ChemBridge 5228409 or ChemBridge 5718127. Comprehensive data summaries from the MSSR pilot screen, primary and counterscreen assays, are provided in Table S1.

### Quality metrics of the S2 primary screening cell line

We determined the quality metrics for HTS for the S2 cell line. We used the ChemBridge 5553825 compound, a potent luciferase inhibitor identified in the pilot screen, to assess quality metrics for S2. ChemBridge 5553825 inhibited CMV-luc in a dose-dependent manner in HEK-293 cells stably transfected with CMV-luc (designated SC cells) without inhibiting cell abundance determined by paired MTT assays (Fig. S2*A*). Using S2 cells, we likewise demonstrated that ChemBridge 5553825 inhibited ATXN2-luc in a dose-dependent manner without altering cell viability (Fig. S2*B*) but not ATXN2-Renilla luciferase (Rluc) (Fig. S2*C*). Collectively, these data are consistent with ChemBridge 5553825 as a firefly luciferase inhibitor rather than an inhibitor of ATXN2-luc or CMV-luc transcription or translation. We then performed 88 replicate luciferase assays per condition in a 384-well plate using S2 cells treated with ChemBridge 5553825 *versus* vehicle (1% dimethyl sulfoxide [DMSO] in phenol red–free Dulbecco’s modified Eagle's medium [DMEM]) (Fig. S2*D*). After 48 h treatment, the luciferase unit means and SDs for cells treated with 1 μM ChemBridge 5553825 or vehicle were 888 ± 149 and 10,321 ± 464, respectively (Fig. S2*E*). Following the study by Zang *et al.* (19), we computed the *Z*′-factor = 0.8 and also determined the signal/noise = 30.0, signal/background = 11.6, and CV = 8.6%.

### National Institutes of Health Chemical Genomics Center pilot screen optimization and miniaturization

We next performed a pilot screen assay to optimize the 1536-well qHTS using S2 cells. A validation screen was performed at five concentrations (ranging from 90 nM to 57 μM) using the LOPAC^1280^ library, a well-characterized set of pharmacologically active molecules. Cytotoxicity was determined using a multiplexed viability assay (CellTiter-Fluor, a fluorescence-based viability assay using Gly-Phe-7-amino-4-trifluoromethylcoumarin substrate). Assay optimizations demonstrated acceptable cellular densities, linear signal increase with cell number plated, and *Z*′-factors >0.7 for any library concentration (Fig. S3). Pilot screening of the LOPAC^1280^ library identified 37 unique active compounds among 58 hits (redundancies occurred) with curve classes between −1.1 and −3 (Table S2). Eleven of the hits were cardiac glycosides (including ouabain and dihydroouabain that were both observed four times), most with curve class −1.1.

### Primary screening assays and counterscreens

Table 1 summarizes the triage of compounds through the screening process. We assayed 363,031 compounds in the 1536-well primary screen using the primary ATXN2-luc S2 cell line assay (libraries described previously). CellTiter-Fluor was used to determine compound effect on cell viability, and Steady-Glo was used to measure luciferase in a multiplexed assay. The *Z*′-scores for the luciferase assays and the multiplexed viability assays for the largest run of 1289 1536-well plates averaged 0.8 (Fig. S4).

**Table 1:** Triage of compounds through pilot and primary screens

Stage	Library	Compounds	Doses^a^	Active (ATXN2-luc)^b^	Passing cytotox triage^c^	Passing CMV-luc triage^d^
MSSR pilot	Multiple^e^	65,728	1	330	12	4
NCATS pilot	LOPAC	1280	5	58	11	11
NCATS primary	MLSMR	357,287	5	1439	757	419
NCATS primary	NPC	2552	5	237	34	n/d
NCATS primary	MIPE	1912	11	503	301	185

Abbreviations: LOPAC, library of pharmacologically active compounds; MIPE, Mechanism Interrogation PlatE; MLSMR, Molecular Libraries Small Molecular Repository; NPC, NCATS Pharmaceutical Collection.

The total number of compounds screened was 428,759.

a MSSR pilot: 10 μM.

b MSSR pilot: compounds lowered ATXN2-luc ≥2.7 SD in H2 cells. Other stages: curve class −1.1, −1.2, −2.1, or −2.2 (with activity score >40) in S2 cells.

c MSSR pilot: Viability reduced by not more than 65% at either 1 or 10 μM in H2 and S1 cells. Other stages: nonactive curve class (values other than −1.1, −1.2, −2.1, or −2.2) in S2 cells.

d MSSR pilot: Less than 30% reduction of CMV-luc in HEK-293 cells stably expressing CMV-luc (designated HC cells) at 10 μM. Other stages: nonactive curve class (values other than −1.1, −1.2, −2.1, or −2.2) in HC cells.

e See Experimental procedures section.

The primary assay of 357,287 compounds from the National Institutes of Health (NIH) Molecular Libraries Small Molecular Repository showed 2763 compounds active against ATXN2-luc in S2 cells, and of these, 2145 had stock solution available for follow-up testing. The 2145 compounds were evaluated in confirmation assays and tested at five concentrations (90 nM–57 μM), showing 1439 compounds confirmed as active against ATXN2-luc in S2 cells, 757 were noncytotoxic, and 416 were inactive against CMV-luc.

The National Center for Advancing Translational Sciences (NCATS) Pharmaceutical Collection (drug repurposing library) of 2552 compounds (20) was also evaluated using a similar process. This screen identified 237 compounds as active in reducing ATXN2-luc levels. Of these 237 compounds, 34 passed the viability counterscreen.

The Mechanism Interrogation PlatE library of 1912 mechanistically annotated small molecules was also screened, which identified 503 compounds as active in reducing ATXN2-luc levels. This library is enriched in oncology-focused compounds (21), so a large number of the 503 compounds were also identified as cytotoxic in the viability counterscreen (202, 40%). Of the 301 remaining nontoxic compounds, 185 were also inactive in the CMV-luc counterscreen. These compounds included several molecular classes and mechanisms of action, including one cardiac glycoside, seven HSP90 inhibitors, three topoisomerase inhibitors, and 10 checkpoint-targeting compounds (cyclin-dependent kinase [CDK]/checkpoint kinase).

Following all the screening efforts, 46 compounds prioritized for further testing, including the HSP90 inhibitors and cardiac glycosides, which appeared as hits from several libraries (Table S3).

### Secondary assays

These 46 compounds were retested at 12 doses and a zero-compound dose, in multiplexed assays in 96-well plates using CellTiter-Fluor to determine cell viability, and then Bright-Glo to determine luciferase expression. The values for H2 and S2 were used as replicates to calculate IC_50_s and to assess cell viability. Among these compounds were 10 HSP90 inhibitors, three NaK-ATPase inhibitors (cardiac glycosides), three topoisomerase inhibitors, seven checkpoint-targeting compounds (CDK, checkpoint kinase, and WEE1 [CDK inhibitor of the Ser/Thr nuclear protein kinase family]), and two casein kinase inhibitors. No other known targeted functions were common among the remaining 21 compounds. Among all the 46 compounds, the two with the lowest IC_50_s were the NaK-ATPase inhibitor proscillaridin A (17 nM) and the HSP90 inhibitor ganetespib (30 nM). Graphs comparing ATXN2-luc, CMV-luc both with separate viability assessments for all the HSP90 inhibitors, and NaK-ATPase inhibitors are provided in Fig. S5.

### Orthogonal assays

For selected compounds targeting NaK-ATPase and HSP90, we verified the ability of compounds to also reduce endogenous *ATXN2* expression in HEK-293 cells.

#### Proscillaridin A

**Figure 2:**
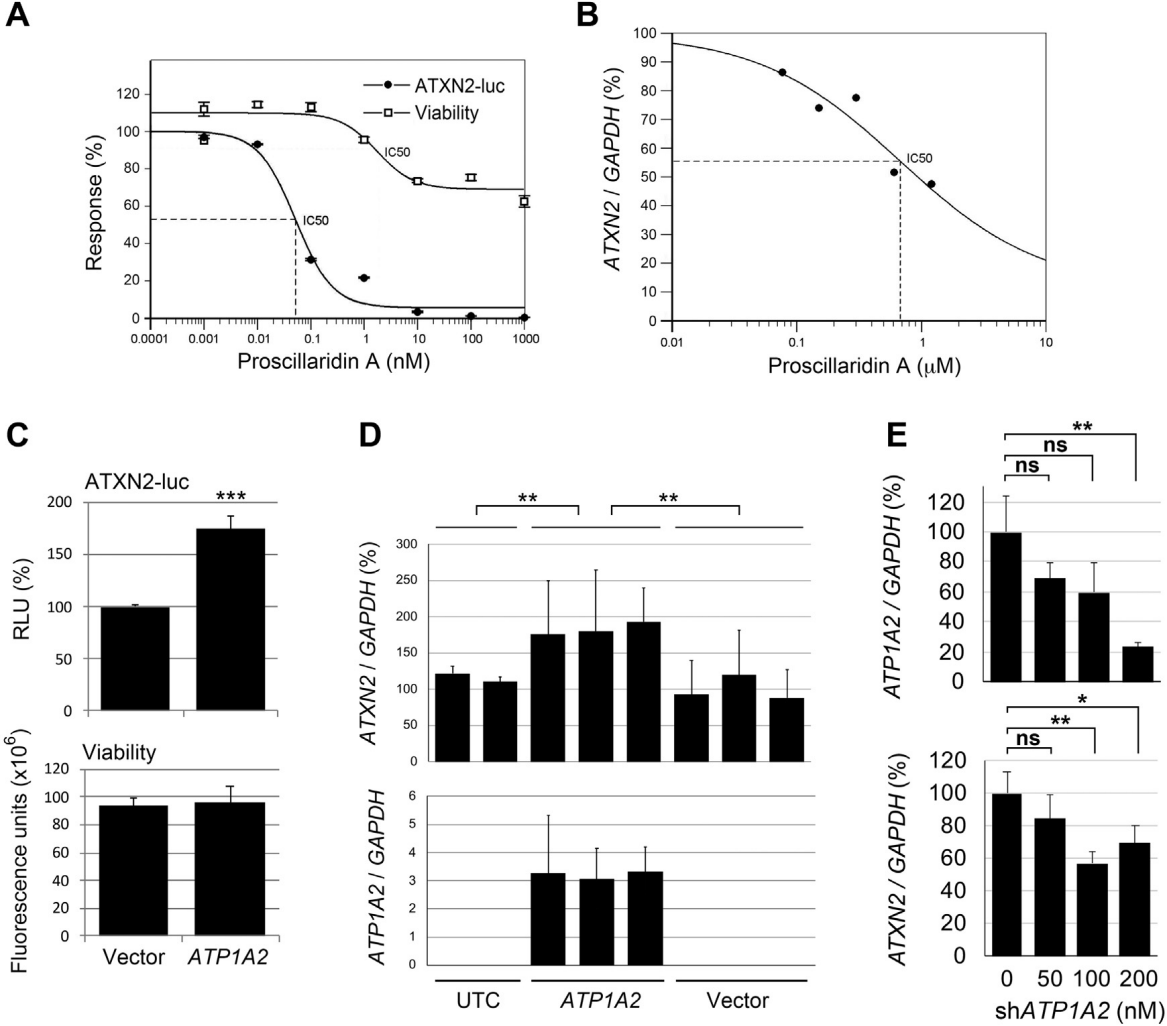
***ATXN2* expression associates with the expression of the proscillaridin A target *ATP1A2*.***A*, H2 were treated 48 h with the indicated doses of proscillaridin A and then evaluated for ATXN2-luc expression by luciferase assay (IC_50_ = 0.052 μM) and for viability by MTT assay. Experiments were repeated three times; values are means and SD. *B*, endogenous *ATXN2* was inhibited better in HEK-293 cells by 48 h of proscillaridin A treatment *versus* 24 h, determined by qPCR (IC_50_ = 0.677 μM). Datapoints represent single values at different doses. *C*, transfection of HEK-293/ATXN2-luc cells (S2) with *ATP1A2* significantly increased ATXN2-luc expression (*top*) but did not alter cell viability determined by CellTiter-Fluor assay (*bottom*). *D*, transfection of HEK-293 cells with *ATP1A2* significantly increased endogenous *ATXN2* expression. Shown are means and SD from three independent transfections, each analyzed by qPCR in triplicates. *E*, lowering *ATP1A2* expression by RNAi significantly reduced *ATXN2* transcription. Statistical tests were Student’s *t* test (*C*) or ANOVA and post hoc Bonferroni corrected *t* tests (*D* and *E*). ∗*p* < 0.05; ∗∗*p* < 0.01; ∗∗∗*p* < 0.001; and ns, not significant. ATXN2, ataxin-2; HEK-293, human embryonic kidney 293 cell line; MTT, 3-(4,5-dimethylthiazol-2-yl)-2,5-diphenyltetrazolium; qPCR, quantitative PCR.

Proscillaridin A had the lowest IC_50_ of any compound tested in the secondary assays, at 17 nM. We reconfirmed dose-dependent reduction of ATXN2-luc in H2 cells with paired viability assays using the MTT method, confirming ATXN2-luc reduction by 80% at 1 nM with a minimal effect on cellular viability (Fig. 2*A*). We then treated HEK-293 cells with increasing doses of proscillaridin A for either 24 and 48 h, showing >50% reduction of endogenous ATXN2 mRNA when cells were treated with 1 nM proscillaridin A at 48 h (Fig. 2*B*). Cardiac glycosides such as proscillaridin A generally target three proteins, ATP1A1, ATP1A2, and ATP1A3 (ATPase Na+/K+ transporting subunit alpha 1/2/3). Of these, ATP1A2 is expressed highly in Purkinje cells (Allen Brain Atlas). When we overexpressed ATP1A2 in H2 cells followed by evaluating ATXN2-luc expression by luciferase assay, the expression of ATXN2-luc was doubled (Fig. 2*C*). Similarly, when we overexpressed ATP1A2 in HEK-293 cells and evaluated *ATXN2* expression by quantitative real-time PCR (qPCR), the expression of ATXN2 was significantly increased (Fig. 2*D*). Finally, we reduced the expression of *ATP1A2* in HEK-293 cells by RNAi resulting in reduced endogenous *ATXN2* transcription, as determined by qPCR (Fig. 2*E*).

#### HSP90 inhibitors

We identified 10 HSP90 inhibitors in our primary screen (ganetespib, luminespib [NVP-AUY922], SNX-5422, SNX-2112, onalespib [AT-13387AU], CNF-2024 [BIIB021], tanespimycin, retaspimycin, KW-2478, and VER-82576 [NVP-BEP800], with IC_50_s ranging from 0.03 to 1.79 μ; Table S3). One additional HSP90 inhibitor was also identified in the MSSR pilot screen, derrubone. This supports HSP90 inhibitors as very effective for lowering *ATXN2* expression; with ganetespib (IC_50_ = 30 nM) exhibiting the second most potent activity for lowering ATXN2-luc expression among the selected compounds (Fig. S5 and Table S3).

**Figure 3:**
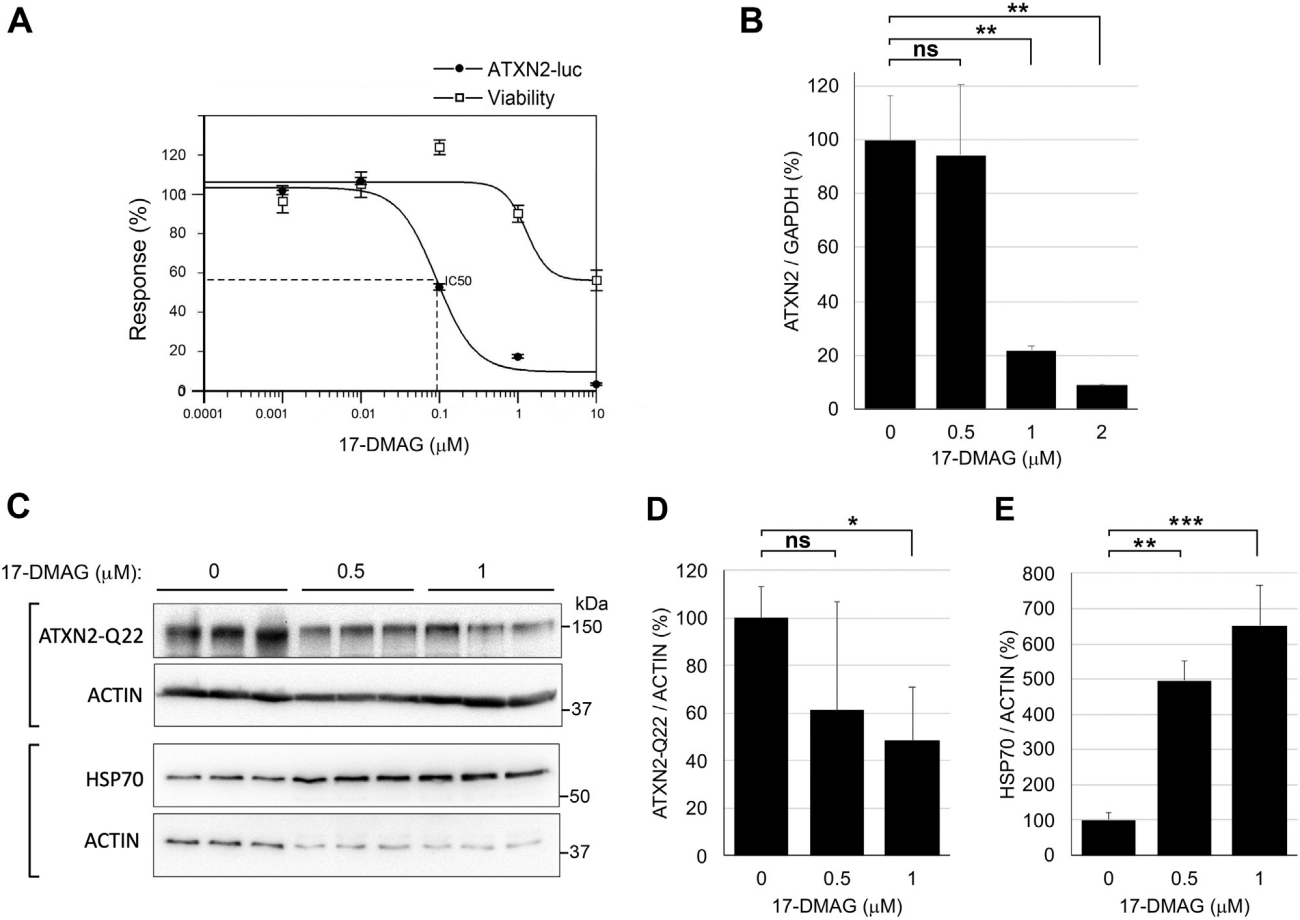
**Effect of HSP90 inhibitor 17-DMAG on ATXN2 expression in HEK-293 cells.***A*, reduction of ATXN2-luc with increasing 17-DMAG doses and effect on cell viability in H2 cells. Cells were treated 48 h with the indicated doses and then evaluated for ATXN2-luc expression by luciferase assay (IC_50_ = 93 nM) and for viability (CellTiter Fluor assay). *B*, 17-DMAG reduced transcription of endogenous ATXN2 in HEK-293 cells in a dose-dependent manner (IC_50_ = 759 nM). *C*, 17-DMAG reduced the expression of endogenous ATXN2 determined by Western blotting in a dose-dependent manner. The *brackets* indicate ACTIN loading controls for the different gels. Experiments were repeated three times; values are means and SD. Statistical tests were ANOVA and post hoc Bonferroni-corrected *t* tests (*D* and *E*). ∗*p* < 0.05; ∗∗*p* < 0.01; ∗∗∗*p* < 0.001. ATXN2, ataxin-2; 17-DMAG, 17-dimethylaminoethylamino-17-demethoxygeldanamycin; HEK-293, human embryonic kidney 293 cell line; HSP90, heat shock protein 90; ns, not significant.

To characterize a bioavailable HSP90 inhibitor, we proceeded by using 17-dimethylaminoethylamino-17-demethoxygeldanamycin (17-DMAG, also known as alvespimycin) for inhibition of ATXN2 in cultured cells instead of the original compounds identified in the screen. We first verified that 17-DMAG treatment of H2 cells resulted in the reduction of ATXN2-luc transcription in H2 cells treated with 1 and 10 μM 17-DMAG for 48 h (Fig. S6). Next, we treated H2 cells with increasing doses of 17-DMAG followed by luciferase assays, which revealed an IC_50_ for lowering ATXN2-luc of 93 nM (Fig. 3*A*). 17-DMAG also reduced the expression of endogenous *ATXN2* transcription in HEK-293 cells in a dose-dependent manner, determined by qPCR, with an IC_50_ of 759 nM (Fig. 3*B*). This pattern parallels that for proscillaridin A, where the IC_50_ for lowering endogenous *ATXN2* transcription in HEK-293 cells that was also an order of magnitude greater than that for lowering ATXN2-luc in the H2 reporter cells, again suggesting the result might be due to a higher ATXN2-luc copy number *versus* endogenous *ATXN2* (see aforementioned). We next showed that 17-DMAG reduced the abundance of endogenous nonmutant ATXN2 protein (Fig. 3*C*) and mutant ATXN2-Q58 protein in HEK-293 knock-in (KI) cells (Fig. 3*D*), determined by Western blotting. We also verified HSP90 target engagement indicated by significantly increased HSP70 abundance (Fig. 3*C*). Inhibition of HSP90 is indicated by upregulation of HSP70, as HSP90 inhibitors block HSP90 interaction with the HSF1 transcription factor, which then forms homotrimers that translocate to the nucleus and transactivate HSP70 and other HSP genes (22, 23).

**Figure 4:**
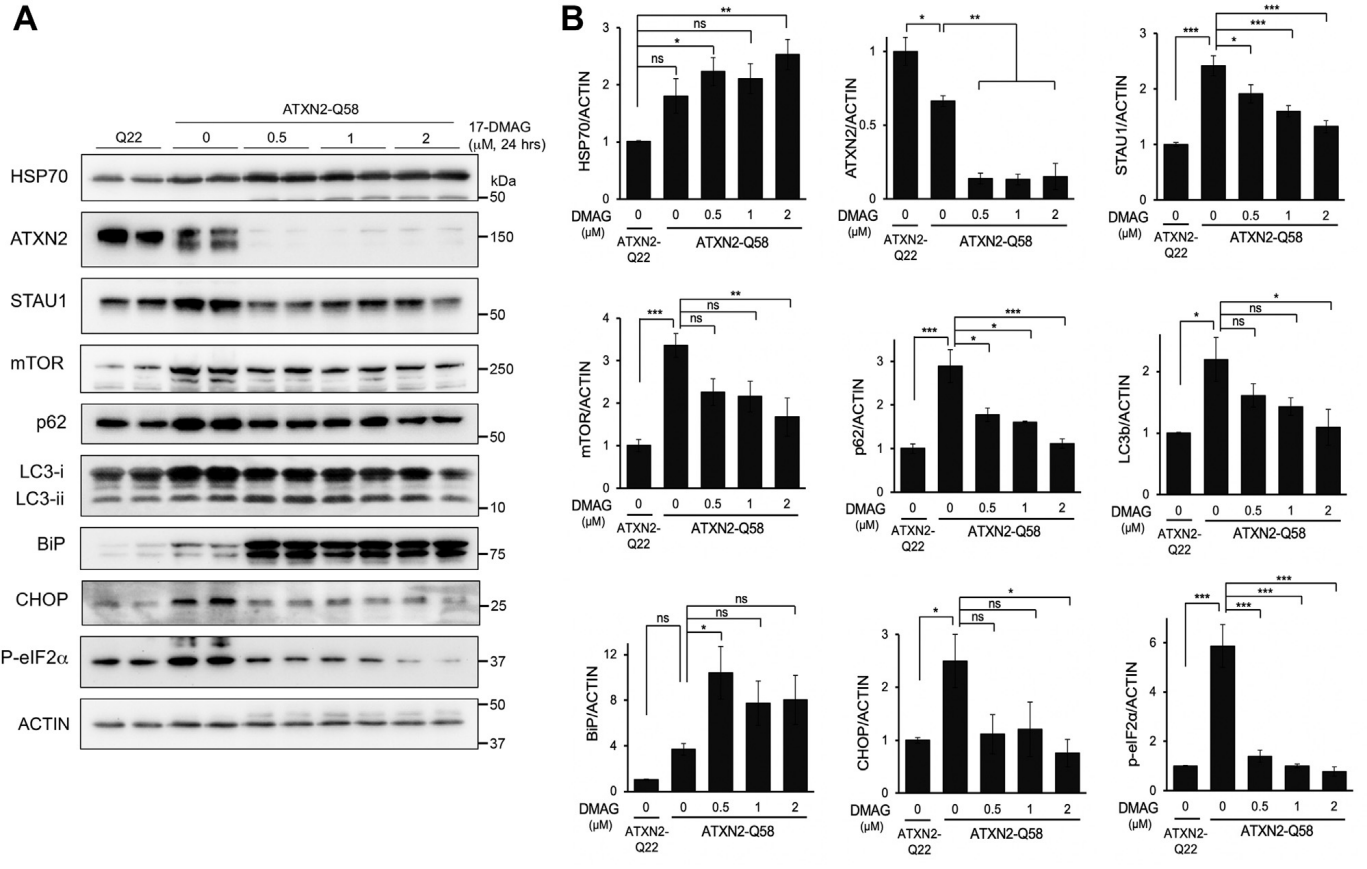
**Reduction of ATXN2 expression by 17-DMAG restores autophagy and ER stress phenotypes in ATXN2 mutant cells.***A*, ATXN2-Q58 HEK-293 cells were treated with 17-DMAG at the indicated doses for 24 h, and then proteins were evaluated by Western blotting. Untreated HEK-293 cells with normal A P-eIF2α TXN2 were also included. Upregulation of HSP70 expression following 17-DMAG is consistent with successful HSP90 target engagement. Reduction of ATXN2 expression by 17-DMAG coincided with normalization of STAU1, p62, mTOR, p62, LC3, BiP, CHOP, and P-eIF2α. Note that the upper ATXN2 band is the human transgenic protein with Q22 and the lower band is the endogenous mouse ATXN2 protein that lacks a polyglutamine tract—these proteins are known to migrate differently. *B*, quantification of the blots in *A*. The n number of blots ranged from 3 to 9. Values are means and SD. Probabilities are from one-way ANOVA and post hoc Bonferroni-corrected *t* tests: ns; ∗*p* < 0.05; ∗∗*p* < 0.01, and ∗∗∗*p* < 0.001. ATXN2, ataxin-2; BiP, binding immunoglobulin protein; CHOP, C/EBP homologous protein; ER, endoplasmic reticulum; 17-DMAG, 17-dimethylaminoethylamino-17-demethoxygeldanamycin; HEK-293, human embryonic kidney 293 cell line; HSP70, heat shock protein 70; LC3, microtubule-associated protein 1A/1B-light chain 3; mTOR, molecular target of rapamycin; ns, not significant; P-eIF2α, phosphorylated eukaryotic initiation factor 2α; STAU1, Staufen 1.

To determine if lowering ATXN2-Q58 in HEK-293 KI cells could restore pathways that we have previously established are abnormal in SCA2 (12, 24), we evaluated several proteins by Western blotting and RNAs by qPCR following 17-DMAG treatment. Again, we observed elevated HSP70 with 17-DMAG treatment, consistent with HSP90 target engagement. When HEK-293 ATXN2-Q58 KI cells were treated with 17-DMAG, we observed significantly reduced mutant ATXN2 protein along with restored abundance of STAU1 and autophagy marker proteins (mTOR, p62, and LC3b [microtubule-associated protein 1A/1B-light chain 3b]), and proteins functioning in the unfolded protein response (UPR) including C/EBP homologous protein (CHOP) and eukaryotic initiation factor 2α (eIF2α), and further elevated binding immunoglobulin protein (BiP; also known as Grp78) (Fig. 4).

**Figure 5:**
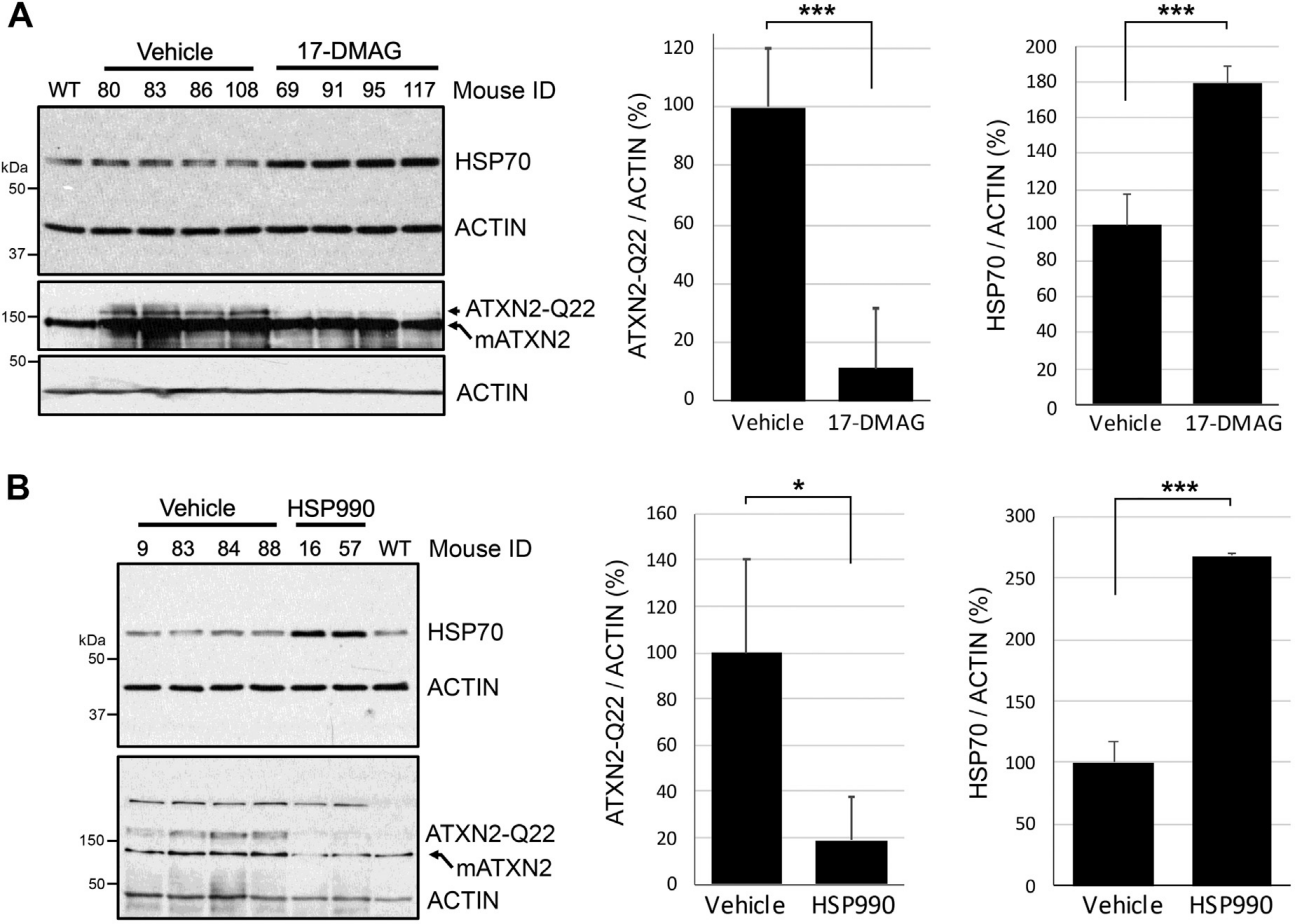
**Effect of HSP90 inhibitors on ATXN2 cerebellar expression *in vivo* in BAC ATXN2-Q22 mice.** Inhibition of ATXN2 expression by 17-DMAG (*A*) and HSP990 (*B*) *versus* vehicle controls. Mouse ID numbers are shown above the lanes. Western blot analysis showing upregulation of HSP70 expression following 17-DMAG treatment, which indicates successful HSP90 target engagement. The human transgenic ATXN2 protein is indicated as ATXN2-Q22 and the endogenous mouse protein as mATXN2. Values shown are mean and SD. Probabilities are from Student’s *t* tests: ∗*p* < 0.05; ∗∗∗*p* < 0.001. Three blots were quantified. ATXN2, ataxin-2; BAC, bacterial artificial chromosome; 17-DMAG, 17-dimethylaminoethylamino-17-demethoxygeldanamycin; HSP, heat shock protein.

We next tested 17-DMAG and another bioavailable HSP90 inhibitor, HSP990, for lowering ATXN2 expression in our human BAC ATXN2-Q22 transgenic mouse model, described previously (14). BAC ATXN2-Q22 mice have all the introns and exons of the human *ATXN2* gene, which expresses a polyQ repeat that is the most common normal allele found in humans. Because ALS might be treated by targeting nonmutant ATXN (5), we did not test HSP90 inhibitors in mice harboring CAG repeat–expanded *ATXN2.* We treated 27-week-old BAC ATXN2-Q22 mice with 17-DMAG (n = 4 per group; two males and two females) every other day over 16 days (days 1, 3, 5, 7, 9, 11, 13, and 15) and then sacrificed on day 16. The expression of ATXN2 and HSP70 was evaluated in cerebellum by Western blotting, which showed 17-DMAG treatment reduced ATXN2 abundance associated with HSP70 induction (Fig. 5*A*). We performed a similar experiment using a second HSP90 inhibitor of a distinct chemotype that can also cross the blood–brain barrier, HSP990. For HSP990, 25-weeks-old BAC ATXN2-Q22 mice were treated (n = 4 per group; two males and two females) every other day over 10 days (days 1, 3, 5, 7, and 9) and then sacrificed on day 10. Unexpectedly, the two females in the HSP990 treatment group did not survive. The expression of ATXN2 and HSP70 was evaluated in cerebellum by Western blotting, and like for 17-DMAG mice, mice treated with HSP990 had reduced ATXN2 abundance associated with HSP70 induction (Fig. 5*B*). Our efforts to evaluate mRNAs from mice treated with 17-DMAG by qPCR were inconclusive because of high variability, likely due to sample quality. However, mice treated with HSP990 had 20% reduced *ATXN2* mRNA abundance in cerebella, determined by qPCR (HSP990-treated group, 78.54 ± 3.9%; vehicle control–treated group, 100 ± 3.16%). The qPCR experiment was replicated by two different technicians, and the *p* value from repeated-measures ANOVA was *p* = 0.0064.

## Discussion

Over the past decade, ATXN2 has emerged as a potential therapeutic target for not only SCA2 but also ALS and other neurodegenerative disorders. CAG repeat expansion mutation in *ATXN2* results in a toxic gain of function for the encoded ATXN2 protein. Lowering ATXN2 expression improved phenotypes in SCA2 and ALS mice (5). In this study, we sought to identify ATXN2-lowering compounds that can serve as scaffolds in the development of small-molecule therapeutics for SCA2 and potentially for ALS.

### Targeting ATXN2 in SCA2 and ALS

Previous work by us indicates ATXN2 as a therapeutic target for both SCA2 and ALS. In SCA2 mice, lowering ATXN2 expression restored the expression of cerebellar proteins and mRNAs, restored Purkinje cell firing frequency, and improved the SCA2 motor rotarod phenotype (18). Targeting *ATXN2* may also be an effective therapeutic approach for treating ALS. Sequencing of *ATXN2* in ALS patients showed that intermediate CAG repeat expansions in *ATXN2* increased ALS risk (4, 25). Hypothetically then, lowering ATXN2 abundance might be an effective approach to treating ALS. The Gitler group tested this hypothesis by crossing a TDP-43 mice with *Atxn2* knockout mice (26), and by lowering *Atxn2* expression in TDP-43 mice using an ASO, demonstrating improved survival and reduced numbers of RNA granules positive for TDP-43 or ATXN2 proteins (5).

We also found that *ATXN2* mutation results in abnormal abundance of autophagy and UPR marker proteins. In cultured SCA2 patient fibroblast cell lines and in cerebellar and spinal cord tissues of SCA2 mouse models, we observed hyperactivated mTOR signaling, resulting in autophagy inhibition marked by increased LC3-II (12). This was attributed to increased mTOR mRNA translation mediated by direct mRNA interaction by the stress-related protein STAU1, which was also highly elevated in these SCA2 cells and models (12, 13). Likewise, in HEK-293 cells expressing mutant ATXN2-Q58, we observed abnormal abundance of proteins in the BiP–protein kinase R-like ER kinase–eIF2α–CHOP pathway indicating UPR activation (27). We now show that STAU1, mTOR-related autophagy proteins, and UPR proteins CHOP and phospho-eIF2α are normalized in ATXN2-Q58 HEK293 cells by 17-DMAG treatment (Fig. 4). Interestingly, we found a significant increase in BiP in response to 17-DMAG. BiP is a key ER chaperone of the HSP70 family that functions as the ER paralog of HSP90 (28, 29). As such, BiP elevation might be compensatory to HSP90 inhibition. Previous reports indicate that its response to HSP90 inhibitors is highly context dependent (30, 31, 32). In ATXN2-Q58 cells, which display basal proapoptotic UPR activation and autophagy dysfunction, the decrease in proapoptotic CHOP associated with a BiP increase could mark a switch to a restorative and adaptive activation of the UPR.

STAU1 abundance and autophagy readouts could be restored by RNAi targeting either *STAU1* or *ATXN2*, and UPR pathways could be restored by RNAi targeting *STAU1* (12, 27). Moreover, we observed elevated STAU1 and hyperactive mTOR signaling with elevated LC3-II in TDP-43 ALS patient fibroblasts and spinal cords of TDP-43 mice, which was restored when mice were haploinsufficient for STAU1 (13). Collectively, these data support that lowering ATXN2 expression can restore abnormal STAU1, autophagy, and UPR signaling associated with disease in SCA2 and ALS. The current study supports that abnormal autophagy and UPR signaling can be improved or normalized using compounds that lower *ATXN2* expression.

### Lowering ATXN2 expression with cardiac glycosides

Many cardiac glycosides were found in our pilot screen. Among the compounds selected following the primary screen, there were three, including proscillaridin A, ouabain, and digoxin, of which proscillaridin A had the lowest IC_50_. Cardiac glycosides are inhibitors of Na^+^/K^+^ ATPases and are used to treat congestive heart failure and cardiac arrythmia. Cardiac glycosides are particularly toxic and able to trigger apoptosis, yet can be antiapoptotic, promoting cellular growth at low doses (33). Overexpression of the Purkinje cell–abundant NaK-ATPase ATP1A2 in HEK-293 cells increased ATXN2 protein abundance (Fig. 2, *C* and *D*), whereas lowering *ATP1A2* expression by RNAi reduced *ATXN2* transcription (Fig. 2*E*). Collectively, these data demonstrate that proscillaridin A and likely other cardiac glycosides regulate ATXN2 abundance transcriptionally, at least in part *via* regulating ATP1A2. More work would be needed to demonstrate that cardiac glycosides lower ATXN2 abundance *in vivo*, as our efforts to accomplish this in a similar way as for HSP90 inhibitors in BAC ATXN2-Q22 mice were unsuccessful.

### Cardiac glycosides for modulating ATXN2 SGs

Lowering ATXN2 expression using cardiac glycosides appears to be an effective strategy for normalizing RNA granules. A hallmark feature of SCA2 is cytoplasmic inclusion bodies in Purkinje cells, and TDP-43/1C2–positive nuclear and cytoplasmic inclusions were observed in SCA2 patient spinal cord motor neurons (34). We have shown that the SG protein STAU1 becomes highly (up to sixfold) elevated in SCA2 and ALS models (12). As described previously, reduction of expression of the nonmutant *ATXN2* gene in TDP-43 models reduced the abundance of ATXN2 and TDP-43–positive SGs (5). In addition, *ATXN2* mutation was associated with increased numbers of TIA-1 (cytotoxic granule–associated RNA-binding protein) positive SGs in HEK-293 cells and in SCA2 patient fibroblasts (12). We have also demonstrated that in SCA2 mice haploinsufficient for *Stau1* that the SCA2 cerebellar molecular phenotype was nearly restored, the motor phenotype was improved, and Purkinje cell inclusion bodies were reduced in numbers (12). A study by Fang *et al.* (35) robustly connected these findings to the regulation of TDP-43–positive SGs by cardiac glycosides. The study identified cardiac glycosides as modulators of SG phenotypes but did not elucidate the molecular mechanism or action *via* the canonical target of cardiac glycoside action. Our data suggest that SG modulation is mediated—at least in part—through reduction of ATXN2 and the ATP1A2 pump. In addition, cardiac glycosides including proscillaridin A can also induce autophagy (36), which might explain their effects on SGs, which are known to be reduced by autophagy (37).

### Lowering ATXN2 expression with HSP90 inhibitors

Our study identified 11 compounds known to inhibit HSP90, including one that was identified in our original pilot screen. Although not identified in our screens, we turned our attention to 17-DMAG (alvespimycin) for *in vitro* experiments because of its prior use in human clinical trials (38) and because 17-DMAG is orally bioavailable and able to cross the blood–brain barrier (39, 40). 17-DMAG inhibited transcription of *ATXN2* in HEK-293 cells in a dose-dependent manner and reduced the abundance of endogenous and mutant ATXN2 proteins in HEK-293 cells (Figs. 3 and 4). Remarkably, BAC ATXN2-Q22 mice treated systemically with 17-DMAG had 90% reduction of ATXN2 protein in the cerebellum (Fig. 5*A*). We also tested another bioavailable HSP90 inhibitor, HSP990, that was also used in a clinical trial but was associated with dose-limiting neurotoxicity (41). Toxicity was evident in our *in vivo* experiment as well. While mice well tolerated 17-DMAG, half of the mice we treated with HSP990 did not survive treatment. However, like for 17-DMAG, BAC ATXN2-Q22 mice treated with HSP990 had 80% reduced ATXN2 protein abundance in the cerebellum (Fig. 5*B*).

Evidence of the possible molecular action of 17-DMAG on ATXN2 expression came from another spinocerebellar ataxia study, on SCA3. ATXN3-135Q transgenic mice treated with 17-DMAG had reduced ATXN3 protein abundance in central nervous system tissues and improved motor coordination, but *ATXN3* mRNA was only moderately reduced (42). ATXN3 inhibition by 17-DMAG was associated with reduced ATXN3 aggregates in the brainstem and evidence for modified autophagic flux (42). 17-DMAG was also effective for clearing aggregates of Huntingtin protein in mammalian cells (43) and for improving polyQ aggregates and motor phenotypes in a spinal-bulbar muscular atrophy mouse model (44). A subset of HSP90 inhibitors including 17-DMAG reduce misfolded proteins by inducing autophagy and can also extend overall health benefits, serving as so-called senolytics (45). These studies raise hope for SCA2, in which HSP90 inhibitors may act dually, by inhibiting *ATXN2* transcription, whereas also modifying autophagic flux to reduce ATXN2 protein abundance and pathogenic aggregates, with other benefits to overall health.

In conclusion, following a qHTS screen of 428,759 compounds, we identified multiple lead compounds including cardiac glycosides and HSP90 inhibitors that lower ATXN2 expression as well as lead compounds acting in other pathways. The identification of these compounds advances understanding on mechanisms involved in ATXN2 expression and protein and aggregate turnover. Compounds selected from the lead list may serve as therapeutics for SCA2 or scaffolds for optimizing SCA2 therapeutics by medicinal chemistry, which may as well treat ALS.

## Experimental procedures

### Plasmid cloning

Plasmid pGL2-5A3 (46) contains 1062 bp of *ATXN2* upstream sequence, the 162 bp *ATXN2* 5′-UTR, and an additional 498 bp of the *ATXN2* exon 1 sequence encoding through the first CAG of the trinucleotide repeat. The construct is useful for reporting *ATXN2* transcription (46). This 498 bp tract includes the start codon at +163 as well as the preferred start codon at +643, located 15 bp upstream of the CAG repeat (see Ref. (46) for our analysis of start codon preference). The firefly luciferase (*luc*) gene follows, in which we substituted the ATG start codon with CTG, followed by the *ATXN2* 3′-UTR (598 bp) and 414 bp of *ATXN2* downstream sequence. A schematic representation of the ATXN2-luc cassette is shown in Figure 1. We modified pGL2-5A3 to include the hygromycin resistance gene. This was accomplished by amplifying the hygromycin resistance gene insert from pTK-HYG (hygromycin; Clontech) with Pfu (*Pyrococcus furiosus*) polymerase (producing blunt ends) using primers HygSalA (5′-CCTCGGTCGACAGCCCAAGCTTGGCACTG-3′) and HygBluntB (5′-CTTGGAGTGGTGAATCCGTTAGCGAGGTG-3′), cutting pGL2-5A3 with SalI and AfeI, and ligating in the hygromycin resistance gene insert prepared by digestion with SalI. The resultant plasmid was designated pGL2h-5A3.

Plasmid pRLh-5A3 is identical to pGL2h-5A3 except *Rluc* (with its ATG start codon substituted to CTG) replaces firefly luciferase. To do so, we first prepared pRL-5A3 by amplifying the *Rluc* insert by PCR from vector pRL-SV40 (Promega) using primers. Renl2-A (5′-GCTACTCGAGCTGACTTCGAAAGTTTATGA-3′) and RenlB (5′-CGCTACCGGTTTATTGTTCATTTTTGAGAA-3′). The amplicon was then digested with XhoI and AfeI and ligated into plasmid pGL2-5A3 prepared by XhoI and AfeI digestion to remove the firefly *luc* insert. pRLh-5A3 with the hygromycin resistance gene was then prepared in the same manner as how the hygromycin gene was inserted into pGL2-5A3, described in the previous paragraph.

Plasmid pGL2h-CMV-luc was prepared using the vector pGL2-Enhancer (Clontech). The CMV insert was amplified from pCMV-MYC (Clontech) with primers CMV-A (5′-GTTGACATTGATTATTGACTA-3′) and CMV-B (5′-GAGCTCTGCTTATATAGA-3′) using Pfu polymerase and ligated into the SmaI site of pGL2-Enhancer, creating the plasmid pGL2-CMV-luc. Plasmid pGL2-CMV-luc was then modified by the addition of the hygromycin resistance gene in the same manner as it was added into pGL2h-5A3, described previously. The resultant plasmid was designated pGL2h-CMV-luc.

All inserts were sequenced in both directions to verify proper plasmid construction.

### Assay cell lines

The primary screening assay cell line was generated by transfecting HEK-293 cells with pGl2h.5A3 and selecting with hygromycin. Transfections were done six separate times resulting in mixed clonal cell lines designated H1, H2, H3, S1, S2, and S3. H2 cells were used in the initial pilot screen. The S2 cell line that expressed a higher level of ATXN2-luc was used in the primary qHTS. Similarly, the counter-screening assay cell line was generated by transfecting HEK-293 cells with pGL2h-CMV-luc and selecting with hygromycin. Two such lines expressing CMV-luc were made, with the resultant cultures designated HC and SC cells. We also generated HEK-293 cells transfected with pRLh-5A3 and selecting with hygromycin, with the resultant cell line designated SR cells. Cultures were maintained as mixed populations of transfected cells (clonal colony selection was not done). Cell lines were tested for the absence of mycoplasma using the Venor GeM *Mycoplasma* Detection Kit (Sigma).

### Libraries and compounds

#### Libraries screened at MSSR

We screened 65,728 compounds from 10 custom and commercially available libraries including compounds from the following sources: Biomol International enzyme inhibitors (300 compounds), Biomol International lipid library (204 compounds), Prestwick Chemicals (1120 compounds), Microsource Spectrum (2000 compounds), Asinex Targeted Library (8505 compounds), Asinex Platinum Collection (19,570 compounds), Biofocus (607 compounds), Chembridge Corp (30,000 compounds), NIH Clinical Collection (606 compounds), and UCLA proprietary ES and S (2816 compounds).

#### Libraries screened at NCATS

A total of 363,031 compounds were screened including compounds in the following libraries: Sigma–Aldrich LOPAC^1280^ (1280 compounds), NIH Small Molecular Repository (Molecular Libraries Small Molecular Repository) (357,287 compounds), NCATS Pharmaceutical Collection (drug repurposing library) (2552 compounds), and NIH Chemical Genomics Center’s mechanistically annotated collection (Mechanism Interrogation PlatE) (1912 compounds).

Other compounds used in the study that were not provided by NCATS or sourced from libraries were the following: 17-DMAG (InvivoGen; catalog no.: ant-dlg-25), HSP990 (Santa Cruz Biotechnology; catalog no.: sc-364508), and proscillaridin A (Sigma; catalog no.: R214310). All compound solutions were prepared in DMSO solvent.

### MSSR pilot screen

The pilot screen was conducted at the UCLA MSSR. We used a reverse plating method with H2 cells in 384-well plates. To do so, 25 μl media were preplated using a Multidrop instrument, 0.5 μl compound was added using a Biomek instrument with a pinning tool, and then 25 μl media containing 5000 cells were added to wells. The final concentration was 10 μM compound in 1% DMSO. Plates were incubated overnight at 37 °C followed by luciferase assays using an automated robotic system for adding 50 μl Bright-Glo reagent and recording of relative light unit values. A total of 197 plates were screened. Using the HTS Corrector software package (47), data were background corrected and normalized by Z-score transformation to create standardized data with mean of 0 and SD of 1 (Z-score = [*X*-μ]/σ where μ and σ are mean and SD, respectively). Compounds lowering luciferase expression by >3 SD were considered positive hits and were “cherry picked” for further analysis. Positive hit compounds were retested at two doses (1 and 10 μM) using H2 cells, S1 cells, and HC cells, with paired MTT viability assays.

### Primary qHTS

The primary assay was conducted at the NCATS NIH Chemical Genomics Center laboratory. About 4 μl of S2 cell suspension in phenol red–free DMEM was dispensed into wells of 1536-well assay plates. After 2 h at 37 °C, compounds were transferred *via* a Kalypsys pin tool equipped with a 1536-pin 23 nl slotted pin array. The majority of assays included final concentrations of 57, 11.4, 2.28, 0.46, 0.091, 0.018, and 0.0037 μM. After incubation at 37 °C for 24 h, Gly-Phe-7-amino-4-trifluoromethylcoumarin (1 μl prepared at 125 μM in PBS) was added, and plates were incubated for 30 min and imaged with a ViewLux high-throughput charged-coupled device imager (PerkinElmer), wherein single end-point fluorescence measurements were acquired to assess cell viability (excitation: 405/10 and emission: 540/25). Next, SteadyGlo luciferase substrate detection reagent (3 μl) was added to each well and incubated for an additional 5 min at room temperature. Luminescence was then measured on the ViewLux imager equipped with clear filters using a 2 s exposure. All screening operations were performed on a fully integrated robotic system (Kalypsys) containing one RX-130 and two RX-90 anthropomorphic robotic arms (Stäubli). Vehicle-only plates, with DMSO being pin-transferred to columns 5 to 48, were inserted uniformly at the beginning and the end of each library in order to monitor and record any shifts in assay performance. Dose–response curves were fit using the Hill equation, and then curve classes were determined as described, where 1 = complete response, 2 = incomplete response, 3 = single point activity, and 4 = inactive (48).

### Primary counterscreen

Compounds evaluated in the primary screen were evaluated in the same manner (viability and luciferase assays) using SC cells expressing CMV-luc. The counterscreen identifies compounds that lower luciferase expression by common transcriptional mechanisms or are luciferase inhibitors. Compounds were considered as active against ATXN2-luc if they inhibited ATXN2-luc in the primary assay (S2 cells) with curve classes −1.1, −1.2, −2.1, or −2.2 and a calculated activity score of >40 (48), did not inhibit CMV-luc in the primary counterscreen (SC cells), and did not alter cell viability in either assay (curve class other than −1.1, −1.2, −2.1, and −2.2 in both assays).

### Recombinant firefly luc inhibitor counterscreen

Confirmed qHTS active compounds were tested in a biochemical assay to directly measure potential for luciferase inhibition using Kinase-Glo (Promega). Briefly, 3 μl of substrate buffer (50 mM Tris–HCl [pH 7.5], 10 mM MgCl_2_, 0.05% bovine serum albumin, 0.01% Tween-20, 13.33 μM d-luciferin (Sigma; catalog no.: L9504), and 13.33 μM ATP) were dispensed into 1536-well assay plates. Compounds were then transferred *via* Kalypsys pin tool equipped with 1536-pin array. Following addition of compound, 1 μl of recombinant firefly luciferase (Sigma; L9506 prepared in 50 mM Tris–HCl [pH 7.5], 10 mM MgCl_2_, 0.05% bovine serum albumin, and 0.01% Tween-20; 10 nM final) was added to initiate the reaction. After 10 min of room temperature incubation, end-point measurements of luminescence were acquired using a ViewLux plate reader equipped with clear filters.

### Secondary assays

Compounds were plated at 2× concentrations in phenol red–free DMEM in 25 μl in 384-well plates with each compound dose plated in triplicate. An equal volume of S2, H2, or SC cells (24,000 cells/well) suspended in phenol red–free DMEM with 10% fetal bovine serum and 1× penicillin/streptomycin were added. Cells were grown 24 h and then assayed first by using the CellTiter-Fluor reagent (20 μl) followed by the addition of Bright-Glo (65 μl) luciferase assay, using a multimode plate reader (Beckman; DT880). Values were reported as the mean ± SD with n = 3. IC_50_s were determined by the Hill coefficient method using an online tool (https://www.aatbio.com/tools/ic50-calculator).

### ATXN2-Q58 KI cell line

ATXN2-Q22/58 KI cells (ATXN2-Q58 KI cells) were generated by CRISPR–Cas9 editing of HEK-293 cells resulting in *ATXN2* alleles with 22 and 58 CAG repeats. The production and characterization of ATXN2-Q58 KI cells was described previously (12).

### Cell line authentication

In order to adhere with the NIH guideline on scientific rigor in conducting biomedical research (NOT-OD-15-103) on the use of biological and/or chemical resources, we authenticated cell lines utilizing shot tandem repeat analysis on 24 loci, including amelogenin for sex identification. The kit used for this was the GenePrint 24 system (Promega).

### RNAi assays

HEK-293 cells were cultured in 6-well dishes in DMEM with 10% fetal bovine serum and 1× penicillin/streptomycin for 24 h. Cells were then transfected with vehicle, 50, 100, or 200 nM of a cocktail of four shRNA plasmids targeting expression of the NaK-ATPase α subunit (Santa Cruz Biochemicals; catalog no.: sc-43956-SH) using Lipofectamine 2000 transfection reagent (Thermo Fisher Scientific).

## Mice

BAC-ATXN2-Q22 (BAC-Q22) mice were previously described (14). BAC-Q22 are transgenic for the complete human *ATXN2* gene with all introns and exons, including 16 kb upstream sequence driving *ATXN2* expression and the complete 3′-UTR. The mice used in this study were maintained on a mixed B6;D2 background with backcrossing to wildtype vendor-purchased (Jackson Laboratories) mice every five generations. Mice were treated with 17-DMAG or HSP990 by intraperitoneal injection, 200 to 300 μl total volume. 17-DMAG was diluted in 40% DMSO and 0.05% Tween-20, and mice received 100 mg/kg 17-DMAG. HSP990 was diluted in 0.6% DMSO and 0.05% Tween-20, and mice received 4 mg/kg HSP990. Control-treated mice included vehicle alone. Mice were treated repeatedly as described in the Results section. Mouse husbandry and surgical procedures were in accordance to Institutional Animal Care and Use Committee–approved protocols.

### Western blotting and antibodies

Proteins were prepared, separated on precast polyacrylamide gels (Bio-Rad), transferred to Hybond (Amersham), and detected by enhanced chemiluminescence (Amersham) as previously described (12, 27). Antibodies included the following: mouse monoclonal anti-ATXN antibody (clone 22/ATXN) (BD Biosciences; catalog no.: 611378), rabbit monoclonal anti-Hsp70 antibody (EPR16892) (Abcam; catalog no.: ab181606), mouse monoclonal anti-β-actin−peroxidase antibody (clone AC-15) (Sigma–Aldrich; catalog no.: A3854), rabbit polyclonal anti-Staufen antibody (Novus Biologicals; catalog no.: NBP1-33202), mouse monoclonal anti-CHOP (L63F7) antibody (Cell Signaling Technology; catalog no.: 2895), rabbit polyclonal antiphospho-eIF2α (Ser51) antibody (Cell Signaling Technology; catalog no.: 9721), rabbit polyclonal anti-mTOR antibody (Cell Signaling Technology; catalog no.: 2972), rabbit polyclonal anti-SQSTM1/p62 antibody (Cell Signaling Technology; catalog no.: 5114), rabbit polyclonal anti-LC3B antibody (Novus Biologicals; catalog no.: NB100-2220), rabbit monoclonal anti-BiP antibody (C50B12) (Cell Signaling Technology; catalog no.: 3177). Secondary antibodies included peroxidase-conjugated horse antimouse immunoglobulin G (Vector Laboratories, catalog no.: PI-2000) and peroxidase AffiniPure goat anti-rabbit immunoglobulin G (Jackson ImmunoResearch Laboratories; catalog no.: 111-035-144).

### qPCR

Total RNA was extracted from cells cultured in 24-well plates using the RNeasy Mini-Kit (Qiagen, Inc) according to the manufacturer’s protocol. DNAse I–treated RNAs were used to synthesize complementary DNA using ProtoScript cDNA synthesis kit (New England Biolabs, Inc). Sets of primers used for qPCR included *ATXN2* primers ATXN2—forward (5′-AAGATATGGACTCCAGTTATGCAAA-3′) and ATXN2—reverse (5′-CAAAGCCTCAAGTTCCTCAT-3′); *ATP1A2* primers ATP1A2—forward (5′-AAGACCCGCCGCAACTCAGTCTTC-3′) and ATP1A2—reverse (5′-ACCAGGTGACTTTGAGCGGGTACA-3′); *GAPDH* primers GAPDH-F133 (5′-GAAGATGGTGATGGGATTTCC-3′) and GAPDH-R333 (5′-GAAGGTGAAGGTCGGAGTCAA-3′); and mouse *Actb* primers Actb–forward (5′-CGTCGACAACGGCTCCGGCATG-3′) and Actb—reverse (5′-GGGCCTCGTCACCCACATAGGAG-3′). qPCR was performed in the Bio-Rad CFX96 instrument (Bio-Rad, Inc) with the Power SYBR Green PCR master mix (Applied Biosystems, Inc). PCR amplification was carried out for 45 cycles. Cycling parameters were denaturation (95 °C for 10 s), annealing (60 °C for 10 s), and extension (72 °C for 40 s). The threshold cycle for each sample was chosen from the linear range and converted to a starting quantity by interpolation from a standard curve run on the same plate for each set of primers.

### Statistical analysis

Two-tailed Student’s *t* tests, repeated-measures ANOVA, or one-way ANOVA followed by Bonferroni's multiple comparisons test were used to determine significant differences among groups. The levels of significance were indicated as follows: ∗*p* ≤ 0.05, ∗∗*p* ≤ 0.01, ∗∗∗*p* ≤ 0.001, ∗∗∗∗*p* ≤ 0.0001 and ns (not significant) = *p* > 0.05. Means ± SDs are presented throughout unless otherwise specified. Statistical tests were carried out using GraphPad Prism, version 9 (GraphPad Software, Inc). In the calculation of mRNA quantities for ATXN2-Q127 mice treated with HSP990, one outlier was removed from the control group that fell >6 SDs from the mean.

## Data availability

All screening data are available in PubChem (AID numbers 588349, 588378, 588380, 651635, 1745845, 1745846, 1745847, 1745848, 1745849, and 1745850).

## Supporting information

This article contains supporting information.

## Conflict of interest

All works by T. D., D. M., and A. J. were performed during employment at the National Center for Advancing Translational Sciences. T. D. is now employed by The Frederick National Laboratory, D. M. by Veralox Therapeutics, Inc, and A. J. by Pfizer, Inc. All other authors declare that they have no conflicts of interest with the contents of this article.
