# Supplementary material for: A quantitative high-throughput screen identifies compounds that lower expression of the SCA2-and ALS-associated gene ATXN2
Source: J Biol Chem. 2022 Jul 2;298(8):102228. doi: 10.1016/j.jbc.2022.102228 (PMC9356275; doi:10.1016/j.jbc.2022.102228)
Supplement: Supporting information figures [file mmc1.pdf]

Supporting Information

Supplementary Figures 1 - 6

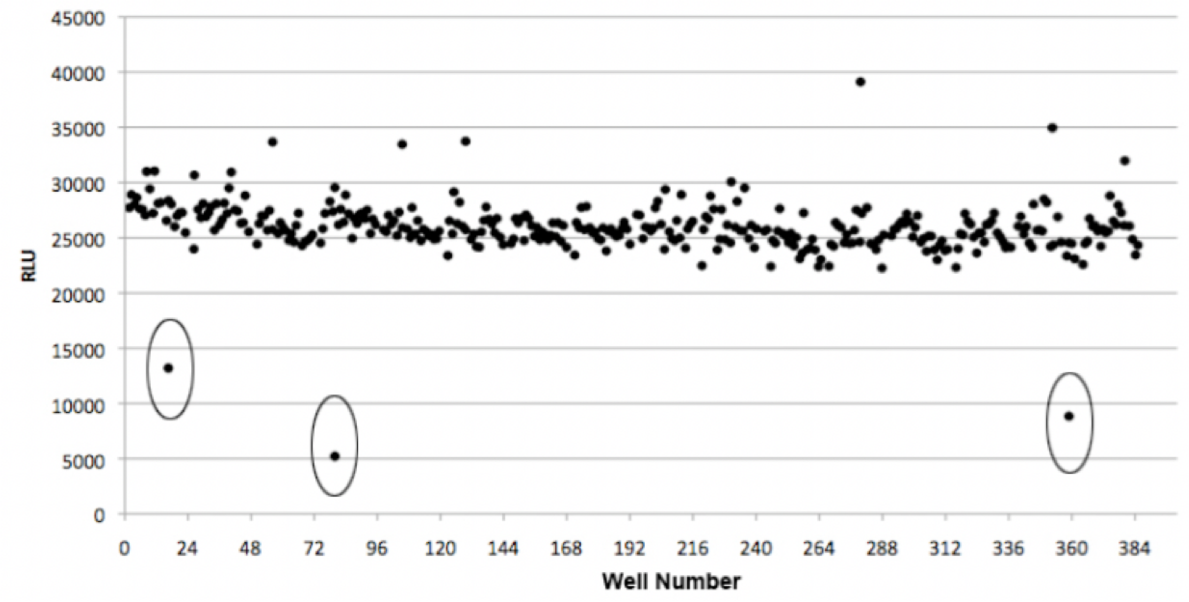

Supplementary Fig. 1. ATXN2-Luc expression (RLU values) for 352 Asinex compounds from one example plate from the pilot MSSR HTS screen. Three hits (circled), from left to right, are > 5, 7, and 6 SD below the mean, respectively.

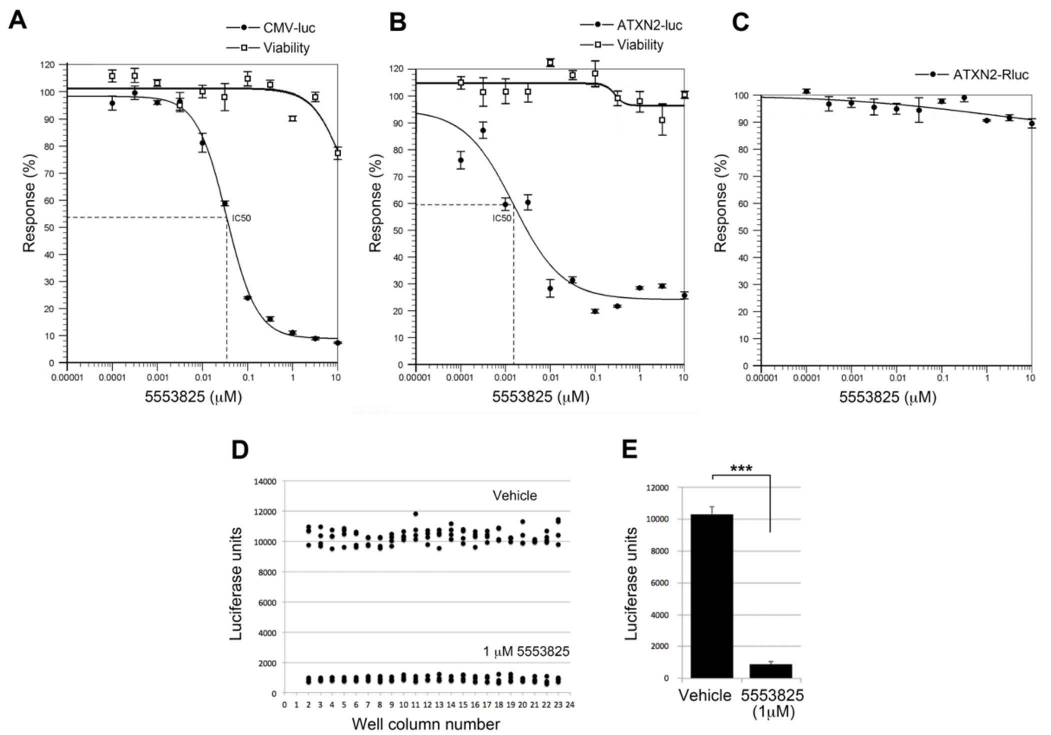

Supplementary Fig. 2. Z'-factor computation for HEK-293/ATXN2-luc cells treated with vehicle vs ChemBridge 5553825 luciferase inhibitor. A-C) ChemBridge 5553825 inhibits luciferase activity. A & B) ChemBridge 5553825 inhibited CMV-luc activity in SC cells (IC50=35 nM) (A) and ATXN2-luc activity in HEK-293/ATXN2-luc cells (S1 cells) (IC50=2 nM) (B), with cellular abundances determined by paired MTT assays. C) ChemBridge 5553825 did not lower activity of ATXN2-Renilla luciferase (ATXN2-Rluc) in stably transfected HEK-293 cells (SR cells). A paired viability assay showed no signal reduction (not shown). These data are consistent with ChemBridge 5553825 as a firefly luciferase inhibitor since it inhibited CMV-luc and ATXN2-luc activities but not ATXN2-Rluc activity. D & E) Z'-factor computation. D) 6000 HEK-293/ATXN2-luc (S1) cells/well were treated with vehicle (1% DMSO) or 1 µM ChemBridge 5553825 in 50 µl total volume in a 384 well plate (n=96 per condition). RLUs were read after 48 hrs. E) Means and SDs for the data in (D) were 888±149 and 10,321±464 luciferase units for 5553825 and vehicle, respectively. ***, $p<0.001$, Student's t-test. The calculated Z'-factor is 0.80. Z'-factor was calculated as $1-[(3(\sigma_{exp}+\sigma_{cont}))/|\mu_{exp}-\mu_{cont}|]$ where σ and µ are SD and mean, respectively.

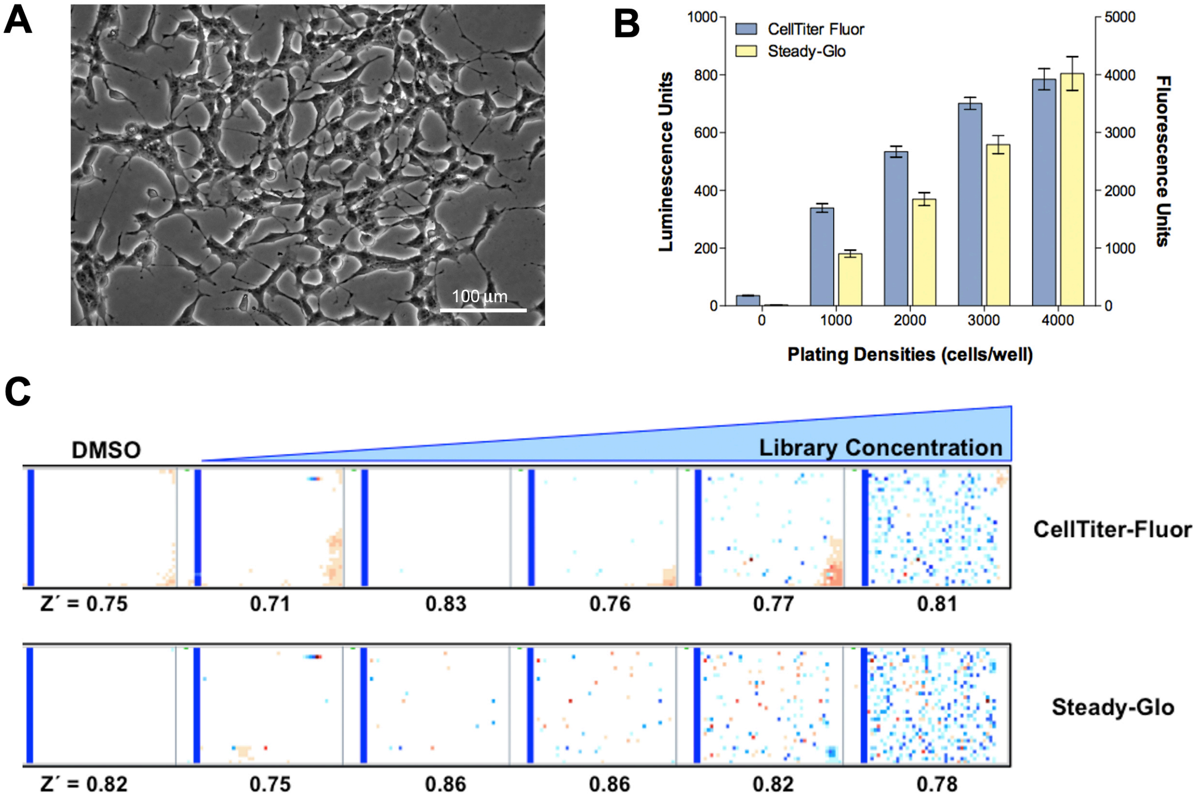

Supplementary Fig. 3. Optimization and miniaturization of the cell-based primary screening assay to 1536 well format, using HEK-293 cells expressing ATXN2-luc (S2 cells). A) S2 cells plated 1000 cells/well in a well of a 1536 well plate showing subconfluent density at time of assay. B) Effect of increasing S2 cell abundance on readout signals. Luciferase expression (Steady-Glo) and a compatible same-well-assay measure of cell abundance (CellTiter Fluor) increased linearly with increasing numbers of cells plated. C) Preliminary miniaturized compound screen using S2 with compound diluent alone (DMSO) or 5 doses of $LOPAC^{1280}$ library compound (Sigma). Cells were plated 1000 cells/well in 1536 well plates. Plates were assayed for ATXN2-luc expression (Steady-Glo) and compound effect on cell abundance (CellTiter-Fluor), with increases shown for each well indicated by red, and decreases indicated by blue. Numerous ATXN2-luc changes were observed for compounds not altering cell abundance. Z'-score values were all > 0.7.

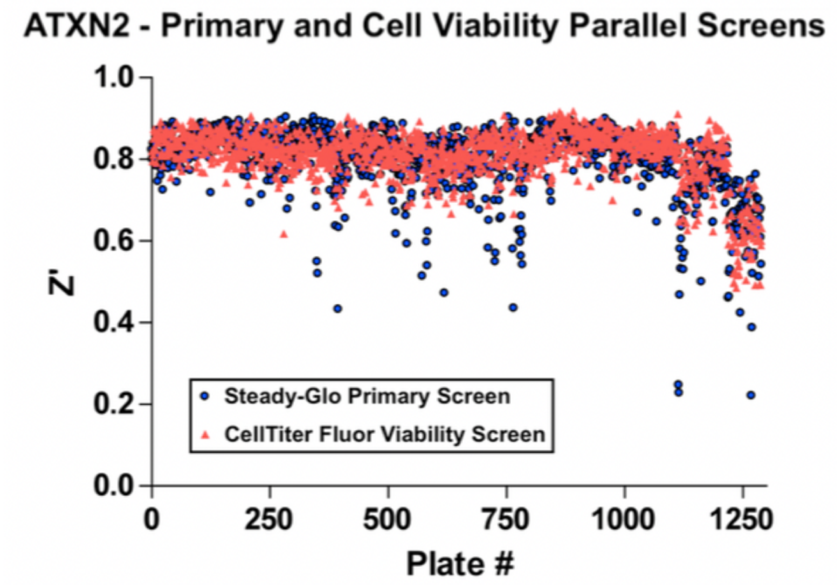

Supplementary Fig. 4. Z'-score vs plate number for 1289 1536-well plates evaluated by qHTS. S2 cells. *Z'* values averaged 0.8.

A. HSP90 inhibitors

Ganetespib, HSP90 inhibitor

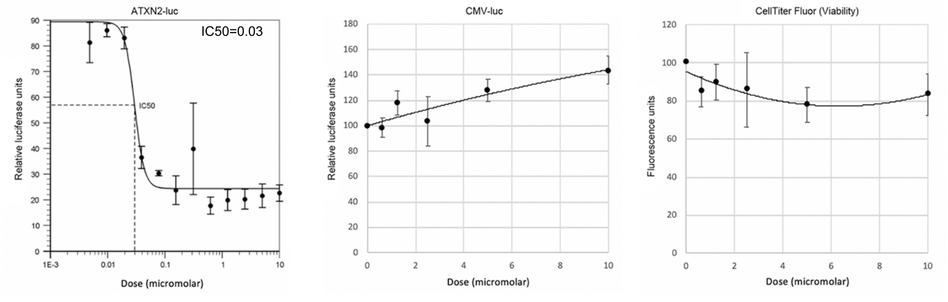

NVP-AUY922, HSP90 inhibitor

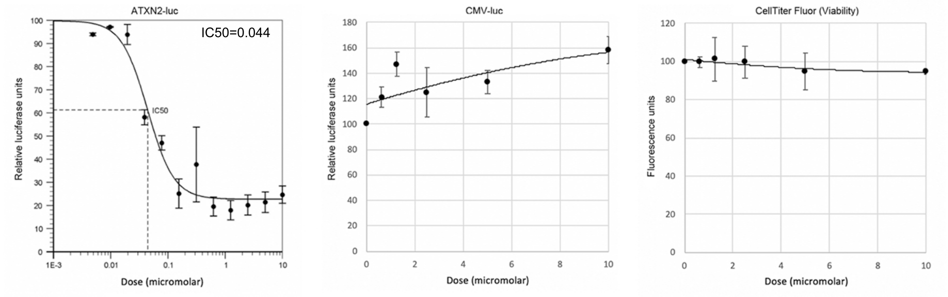

SNX-5422, HSP90 inhibitor

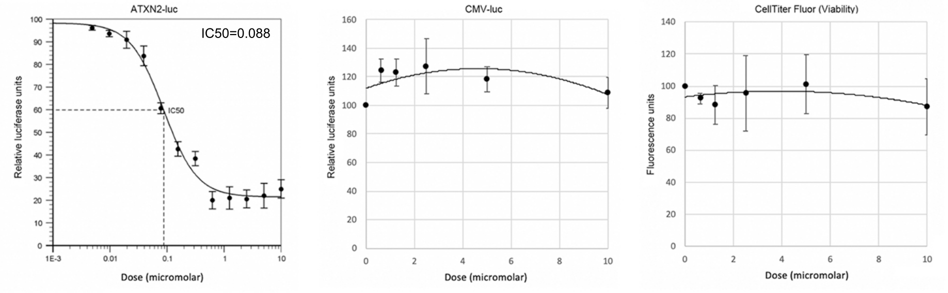

SNX-2112, HSP90 inhibitor

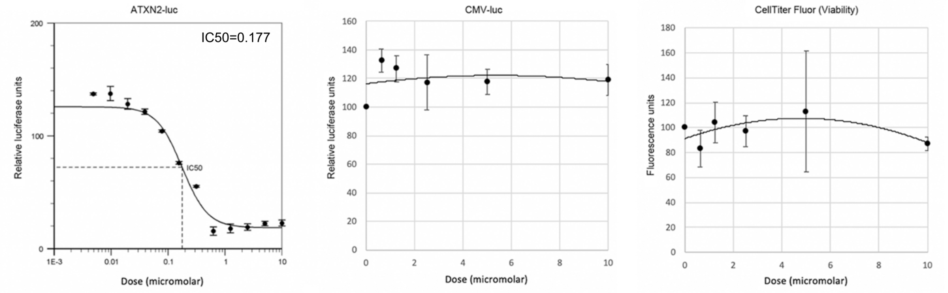

AT-13387AU, HSP90 inhibitor

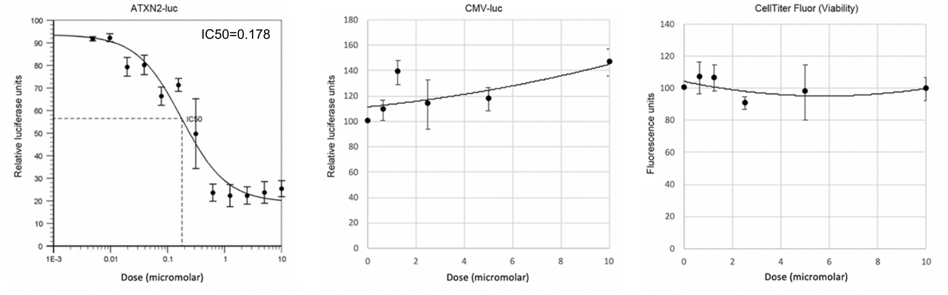

Supplementary Fig. 5 (page 1 of 3)

A. HSP90 inhibitors, cont.

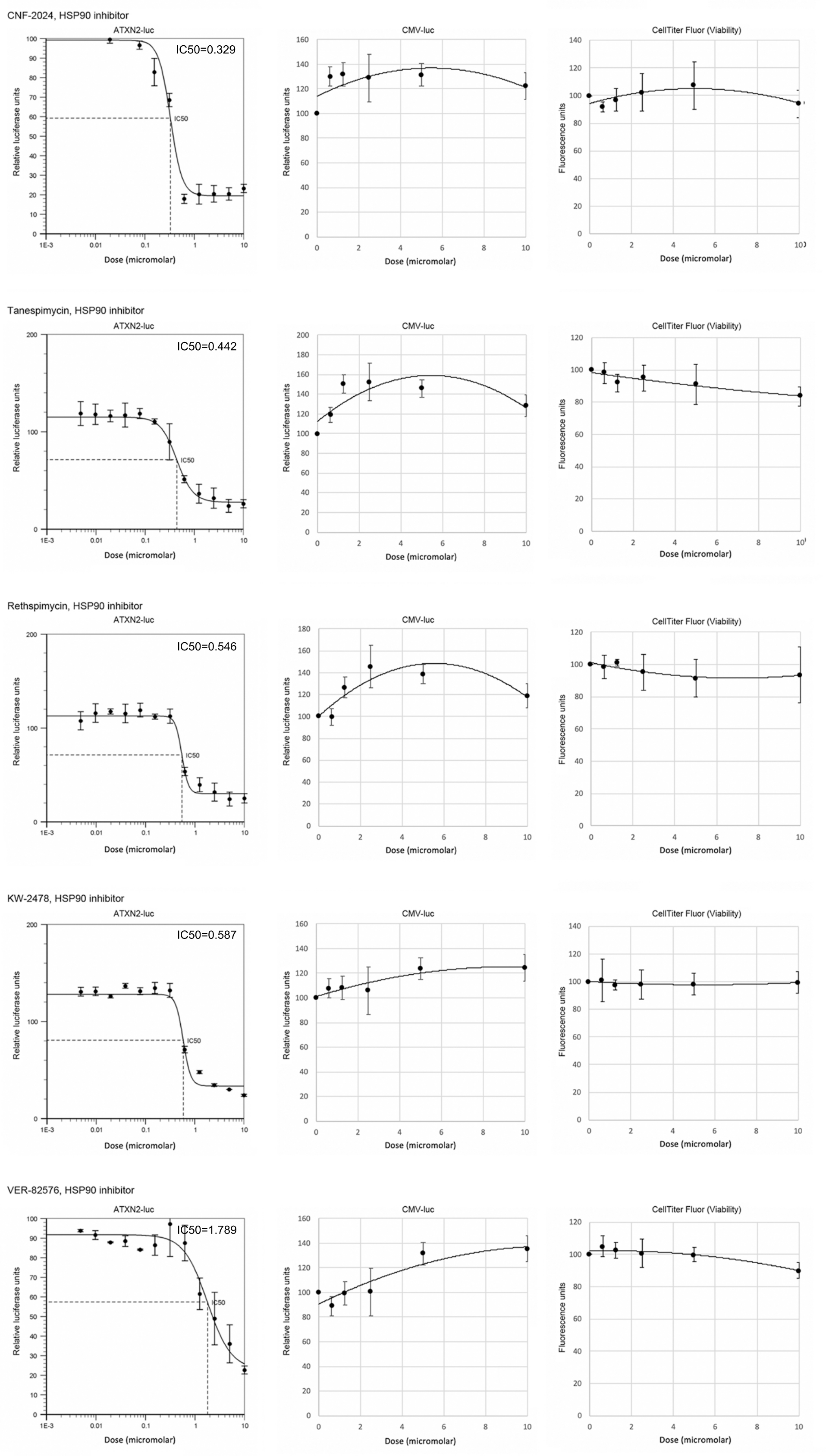

Supplementary Fig. 5 (page 2 of 3)

B. NaK-ATPase inhibitors.

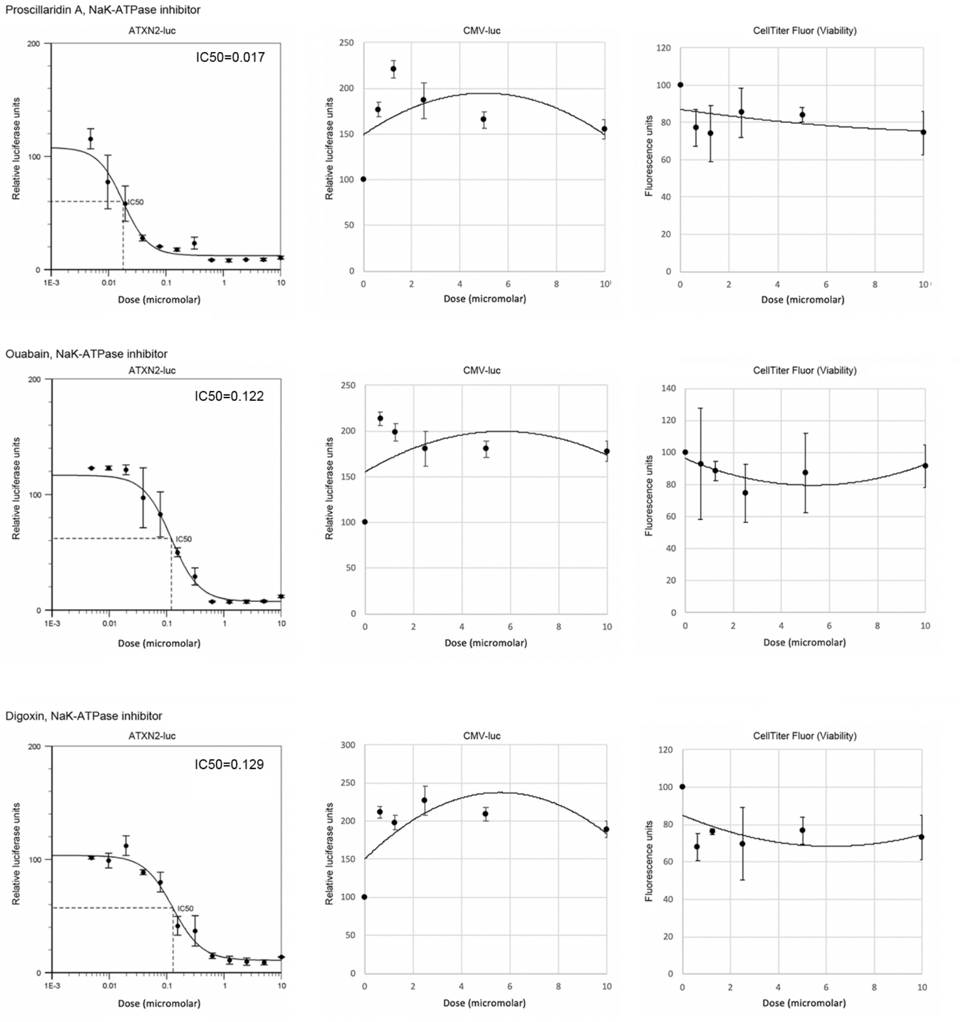

Supplementary Fig. 5. Top HSP90 inhibitors (A) and NaK-ATPase inhibitors (B), ranked by IC50. Left: Relative luciferase units (RLUs) averaged for H2 and S2 cells expressing ATXN2-luc (n=2 cell lines). The fitted line was determined using the Hill coefficient method for determining IC50s, shown on the charts. Center: RLUs for SC cells expressing CMV-luc (n=1 cell line). Right: Average viability for H2, S2 and SC cells determined using CellTiter Fluor (n=3). For CMV-luc and viability charts, the fitted lines are second-order polynomials. Means and SD are shown.

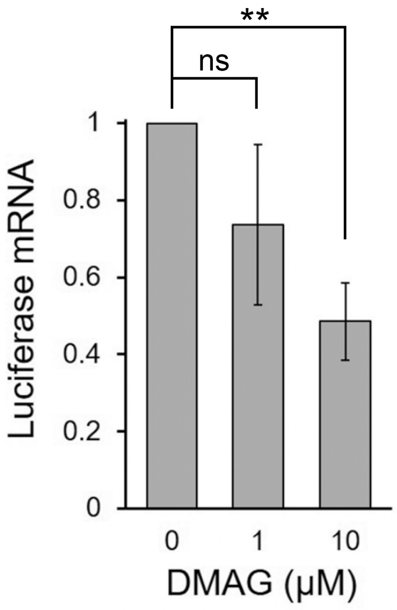

Supplementary Fig. 6. DMAG reduces transcription of ATXN2-luc in H2 cells. H2 cells were treated with the indicated doses of DMAG for 48 hours, then ATXN2-luc mRNA abundance was determined by quantitative PCR using primers that amplify the luciferase gene. Probablities were determined by one-way ANOVA and post-hoc Bonferroni corrected t-tests: ns, not significant; **, $P<0.01$.
